# Dietary Restriction Ameliorates Diabetic Nephropathy through Anti-Inflammatory Effects and Regulation of the Autophagy via Restoration of Sirt1 in Diabetic Wistar Fatty (*fa/fa*) Rats: A Model of Type 2 Diabetes

**DOI:** 10.1155/2011/908185

**Published:** 2011-09-22

**Authors:** Munehiro Kitada, Ai Takeda, Takako Nagai, Hiroki Ito, Keizo Kanasaki, Daisuke Koya

**Affiliations:** Division of Diabetes & Endocrinology, Kanazawa Medical University, Kahoku-Gun, Ishikawa Prefecture 920-0293, Japan

## Abstract

*Aim*. Despite the beneficial effects of dietary restriction (DR) on lifespan, age-related diseases, including diabetes and cardiovascular diseases, its effects on type 2 diabetic nephropathy remain unknown. This study examined the renoprotective effects of DR in Wistar fatty (*fa/fa*) rats (WFRs). *Methods*. WFRs were treated with DR (40% restriction) for 24 weeks. Urinary albumin excretion, creatinine clearance, renal histologies, acetylated-NF-**κ**B (p65), Sirt1 protein expression, and p62/Sqstm 1 accumulation in the renal cortex, as well as electron microscopic observation of mitochondrial morphology and autophagosomes in proximal tubular cells were estimated. *Results*. DR ameliorated renal abnormalities including inflammation in WFRs. The decrease in Sirt1 levels, increase in acetylated-NF-**κ**B, and impaired autophagy in WFRs were improved by DR. *Conclusions*. DR exerted anti-inflammatory effects and improved the dysregulation of autophagy through the restoration of Sirt1 in the kidneys of WFRs, which resulted in the amelioration of renal injuries in type 2 diabetes.

## 1. Introduction

According to the International Diabetes Federation (IDF) atlas in 2009, the estimated diabetes prevalence for 2010 has risen to 285 million, representing 6.6% of the world's adult population, with a prediction that by 2030 the number of people with diabetes will have risen to 438 million [1]. Diabetes results in vascular changes and dysfunction, and diabetic complications are the major cause of morbidity and mortality in diabetic patients. Among diabetic vascular complications, nephropathy is a leading cause of end-stage renal disease and an independent risk factor for cardiovascular diseases. Renal inflammation is recognized as one of the important pathophysiological mechanisms and therapeutic targets for the prevention of diabetic nephropathy and atherosclerosis [2]. 

Dietary restriction (DR) has a variety of effects on lifespan extension and the delayed onset of age-related diseases, including cardiovascular diseases and diabetes, and is accepted as the only established antiaging experimental paradigm [3, 4]. The beneficial effects of DR involve the function of the NAD^+^-dependent deacetylase, Sirt1, the expression of which is induced by DR. Sirt1 has cytoprotective effects, including anti-inflammatory effects [5–7]. 

In addition, autophagy is a major intracellular process in which lysises in sthe damaged cytoplasmic organelles, including mitochondria, degrade protein aggregates and aged proteins [8, 9]. Although the renoprotective effects of autophagy have been shown in several animal experimental models such as aging [10] and acute kidney injury [11–14], the role of autophagy in diabetic nephropathy remains unknown. Moreover, Sirt1 is one of the positive regulators of autophagy [15]. However, there is little information on whether DR ameliorates nephropathy in type 2 diabetes, and if so, whether the effects of DR are associated with Sirt1 through anti-inflammatory function or through the regulation of autophagy. 

Therefore, the aim of this study was to investigate the potential effects of DR on inflammation and the regulation of autophagy in the diabetic kidney. We demonstrated that DR attenuated inflammation related to the increase in acetylated-NF-*κ*B and the dysregulation of autophagy as a result of normalized mitochondrial morphologies through restoration of Sirt1 expression in the kidneys of WFRs.

## 2. Research Design and Methods

### 2.1. Materials and Antibodies

The anti-Sirt1 antibody was purchased from Millipore (Billerica, Mass). The anti-phospho-AMPK*α* (Thr172) and anti-AMPK*α* (23A3) antibodies were obtained from Cell Signaling Technology (Beverly, MA). The acetylated-NF-*κ*B (p65, K310) and NF-*κ*B (p65) antibodies were purchased from Abcam (Cambridge, MA). The anti-ED-1 antibody was purchased from Serotec (Oxford, UK). The anti-p62/Sqstm1 antibody was obtained from Medical & Biological Laboratories (Nagoya, Japan). The Rat Microalbuminuria ELISA kit (NEPHRAT II) was purchased from Exocell (Philadelphia, P).

### 2.2. Animals

Male diabetic Wistar fatty (*fa/fa*) rats (WFRs), a model of type 2 diabetes, and age-matched nondiabetic Wistar lean rats (WLRs) were obtained from Takeda Chemical Industries (Osaka, Japan) [16]. At 6 weeks of age, rats were divided into four groups: WLRs, WFRs and WLRs treated with DR, and WFRs treated with DR. The DR group was given a daily 40% restriction of the food consumption of WLRs or WFRs control rats. Food consumption was measured twice a week. Body weight and blood glucose levels were measured every two weeks in all animals. The blood pressure of conscious rats was measured at steady state using a programmable tail-cuff sphygmomanometer (BP98-A; Softron, Tokyo, Japan) once a month. After 24 weeks, individual rats were placed in metabolic cages for 24 h urine collection. The urine samples were stored at −80°C until analysis. Rats were anesthetized by an intraperitoneal injection of sodium pentobarbital, and the right kidneys were removed and stored at −80°C for the experiments described below. After the collection of blood samples from the left cardiac ventricle, the left kidney was perfused with ice-cold phosphate-buffered saline (PBS) and 10% neutral-buffered formalin and removed. The Research Center for Animal Life Science of Kanazawa Medical University approved all experiments.

### 2.3. Blood and Urinary Analysis

Glycated-Hb levels were measured using a DCA 2000 analyzer (Siemens Medical Solutions Diagnostics, Tokyo, Japan). Triglycerides (TG) and total cholesterol (T-CHO) were measured using a Pure-Auto S TG-N kit (Sekisui Medical, Tokyo, Japan) and an L-type cholesterol *H*-test kit (Wako Pure Chemical Industries, Osaka, Japan), respectively. Serum creatinine was measured using a Cica liquid-S CRE kit (Kanto Chemical Co., Inc, Tokyo, Japan), and urinary creatinine concentration was measured with a BioMajesty JCA-BM12 (Hitachi, Tokyo, Japan). Urinary albumin excretion (UAE) was measured using an enzyme-linked immunosorbent assay (ELISA) kit, and the results are expressed as the total amount of albumin excreted in 24 h urine collection. Body weight-adjusted creatinine clearance (Ccr) was calculated with the following equation: Ccr = urine creatinine (mg/dL) × urine volume (*μ*L/min)/serum creatinine (mg/dL)/body wt (g) [17].

### 2.4. Morphological Analysis and Immunohistochemistry

Paraffin sections (3 *μ*m thick) of the fixed and processed kidneys were stained with periodic acid/Schiff (PAS) reagent or Masson-Trichrome. To assess the mesangial expansion, 20 glomeruli, that were randomly selected from each rat were cut at the vascular pole, and the periodic acid/Schiff- (PAS-) positive material in the mesangial area and glomerular tuft area was measured using computer-assisted color image analysis (Micro Analyzer; Japan Poladigital, Tokyo, Japan) as described previously [18]. For the semiquantitative evaluation of fibrosis by Masson-Trichrome staining in the kidney, 20 randomly selected glomerulus or tubulointerstitial areas per rat were graded in a double-blind manner, as reported previously, with minor modifications [10]. 

Immunohistochemical staining of 3 *μ*m paraffin sections was performed as described previously [18]. In brief, the sections were deparaffinized and rehydrated by incubating at room temperature in xylene three times for 3 min, in 100% ethanol twice for 3 min, in 95% ethanol twice for 3 min, and once in Tris-buffered saline (TBS; 0.1 mol/L Tris-HCl, pH 7.4, 0.15 mol/L NaCl) for 5 min. The sections were immersed in 3% H_2_O_2_ absolute methanol solution at room temperature for 15 min. After washing with TBS, sections were incubated overnight at 4°C with primary antibodies raised against ED-1 (1 : 50), followed by amino acid polymers that are conjugated to multiple molecules of peroxidase and antimouse IgG and washed with TBS. The sections were stained with ACE solution for 20 min at room temperature and washed with distilled water, followed by counterstaining with hematoxyline. For quantitative analysis of ED-1 staining, ED-1-labeled cells in the 20 randomly selected glomeruli and interstitial areas of the renal cortex were counted per each animal and analyzed individually as described previously [18, 19]. An investigator who was blinded to the sample identity performed the image analysis.

### 2.5. Western Blot Analysis

The renal cortex sample was homogenized in ice-cold RIPA buffer. Samples of protein solutions from the kidney were used for Western blotting. These samples were separated on 15% SDS-PAGE gels and transferred to a polyvinylidene difluoride filter (Immobilon; Millipore, Bedford, MA). After blocking with 5% milk, the filter was incubated overnight with an anti-acetylated-NF-*κ*B (p65) (1 : 1000), NF-*κ*B (1 : 1000), p62/Sqstm1 (1 : 1000), or Sirt1 antibody (1 : 1000) at 4°C. The filter was then incubated with the appropriate HRP-conjugated secondary antibodies (Amersham, Buckinghamshire, UK), and the bands were detected by enhanced chemiluminescence (Amersham, Buckinghamshire, UK).

### 2.6. Quantitative RT-PCR

The isolation of total RNA from the renal cortex and the determination of cDNA synthesis by reverse transcription and quantitative real-time PCR were performed as described previously [14]. The PCR primer sets are listed below. TGF-*β*: sense (5′-3′) TGCGCCTGCAGAGATTCAAG, antisense (5′-3′) AGGTAACGCCAGGAATTGTTGCTA, fibronectin: sense (5′-3′) GCACATGTCTCGGGAATGGA, antisense (5′-3′) ACACGTGCAGGAGCAAATGG, collagen IV: sense (5′-3′) GTGTCAGCAATTAGGCAGGTCAAG, antisense (5′-3) CTGGTGTTGGAAACCCTGTGAA, MCP-1: Sense (5′-3′) CTATGCAGGTCTCTGTCACGCTTC, antisense (5′-3′) CAGCCGACTCATTGGGATCA, ICAM-1: sense (5′-3′) ACAAGTGCCGTGCCTTTAGCTC, Anti-sense (5′-3′) GATCACGAAGCCCGCAATG, VCAM-1: sense (5′-3′) GGATGCCGGAGTATACGAGTGTG, antisense (5′-3′) CAATGGCGGGTATTACCAAGGA, 18S: Sense (5′-3′) TTCCGATAACGAACGAGACTCT, Anti-sense (5′-3′) TGGCTGAACGCCACTTGTC.

### 2.7. Electron Microscopy

Part of the harvested kidney was cut into small tissues blocks (1 mm^3^) and fixed in 2% glutaraldehyde in 0.1 M potassium phosphate sodium buffer at 4°C for examination by electron microscopy. After postfixation with 2% osmium tetroxide, tissues were dehydrated in a series of graded ethanol solutions. Ethanol was then substituted for propylene oxide, and the samples were embedded in epoxy resin. Ultrathin sections were double stained with uranyl acetate and lead citrate. Sections were examined using a JEM1200EX electron microscope (JEOL, Tokyo) at 80 keV. The mitochondria morphology and autophagosomes in the proximal tubular cells were observed by electron microscopy as described previously [10, 14]. 

### 2.8. Statistical Analysis

Data are expressed as means ± SD. The Tukey multiple-comparison test was used to determine the significance of pairwise differences among three or more groups. *P* < 0.05 was considered significant. 

## 3. Results

### 3.1. Characteristics of Experimental Rats

The characteristics of the four groups of rats at the end of the experimental period were shown in Table 1. The whole body and kidney weights were significantly higher in WFRs than in the other group. The systolic blood pressure (SBP) was not significantly changed in any groups. The WFRs exhibited elevated fasting blood glucose levels and glycated-Hb compared to WLRs, and DR induced a partial improvement in glycated-Hb levels by the end of the experimental period. Serum lipid profiles including T-CHO and TG levels were also significantly elevated in WFRs compared to WLRs; the increases in T-CHO and TG levels were partially rescued by DR (Table 1). 

### 3.2. Changes in Urinary Albumin Excretion and Creatinine Clearance (Ccr)

**Figure 1:**
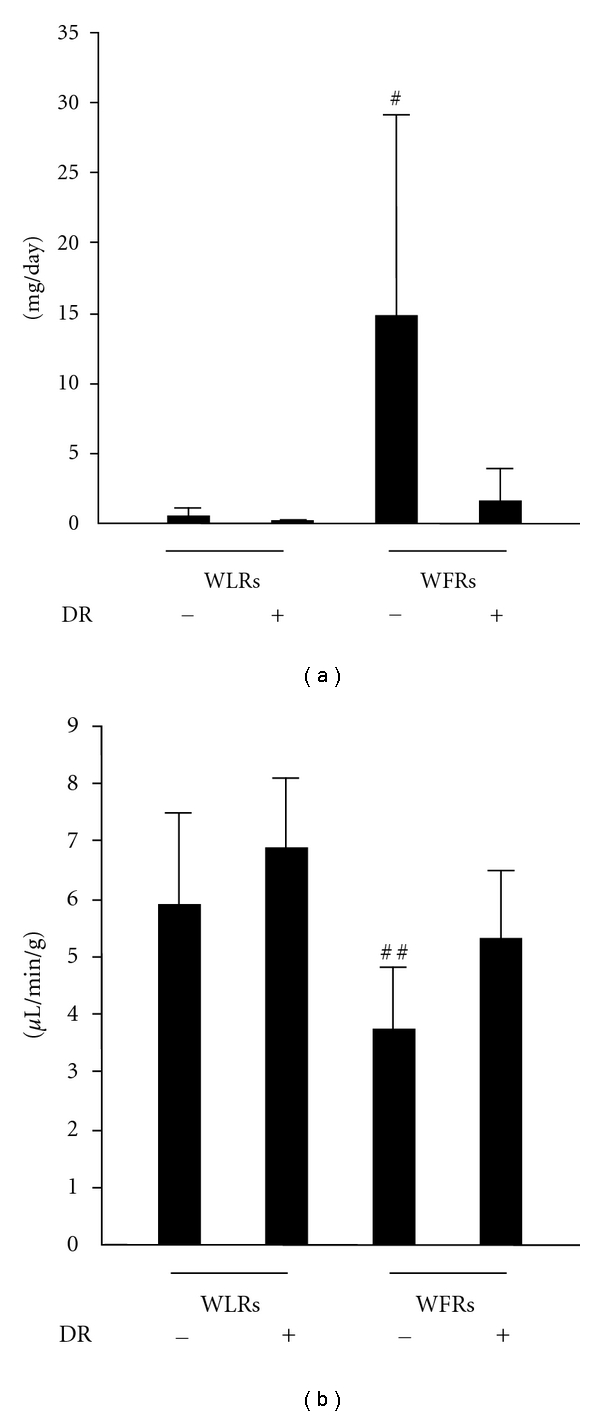
DR ameliorates urinary albumin excretion (a) and creatinine clearance (Ccr) (b) in Wistar fatty rats (WFRs). Treatment with DR reduced urinary albumin excretion and improved Ccr in WFRs. Data are means ± SD (*n* = 9 − 11, ^#^ *P* < 0.01 versus other groups, ^##^ *P* < 0.05 versus other groups).

To evaluate the effects of DR on renal dysfunction in WFRs, we measured the urinary albumin excretion and Ccr. The values for urinary albumin excretion were higher, and the Ccrs were lower in WFRs than those in WLRs. Treatment with DR significantly reduced the urinary albumin excretion and restored Ccr, which indicated that DR ameliorated the functional abnormalities of nephropathy in WFRs (Figures 1(a) and 1(b)).

### 3.3. Changes in Kidney Morphology

**Figure 2:**
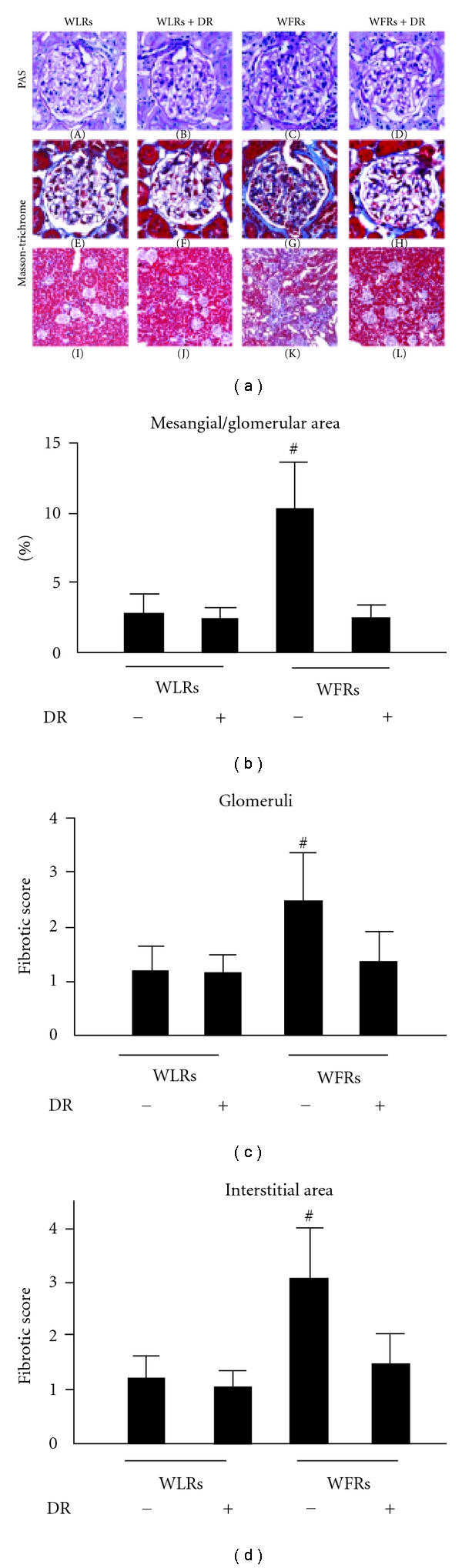
DR ameliorates mesangial expansion and renal fibrosis in WFRs. ((a)-(A) through (D)) Representative photomicrographs of PAS-stained kidney sections from four groups of rats. Data are the results of independent experiments in each group with six rats per group. Original magnification, ×400. (b) Quantitative assessment of the mesangial matrix area in the four groups of rats. Data are means ± SD. (*n* = 6, ^#^ *P* < 0.01 versus other groups). Treatment with DR reduced glomerular and interstitial fibrosis in WFRs. ((a)-(E) through (H), (I) through (L)) Representative photomicrographs of Masson-Trichrome staining in the four groups of rats. Data are the results of independent experiments in each group with six rats per group. Original magnification, ×400 for glomerular fibrosis and ×100 for interstitial fibrosis. (c) and (d) Quantitative assessment of fibrosis in the four groups of rats. Data are means ± SD (*n* = 6, ^#^ *P* < 0.01 versus other groups).

Representative photomicrographs of mesangial matrix accumulation in the PAS-stained kidneys of the four groups were shown in Figure 2(a)—(A)–(D). The results of the quantitative analysis of mesangial matrix expansion in all groups were shown in Figure 2(b). Although the ratios of mesangial matrix/glomerular area were significantly larger in WFRs than that in WLRs, DR significantly ameliorated the mesangial expansion in WFRs. 

The renal fibrosis determined by Masson-Trichrome staining (Figure 2(a)—(E) through (H) and (I) through (L)) also revealed a significantly higher score for renal glomerular and tubulointerstitial lesions in WFRs than those in WLRs (Figures 2(c) and 2(d)). DR reduced the increase in score for Masson-Trichrome staining in WFRs (Figures 2(c) and 2(d)). 

### 3.4. mRNA Levels of TGF-*β*1, Fibronectin, and Collagen IV in the Kidney

**Figure 3:**
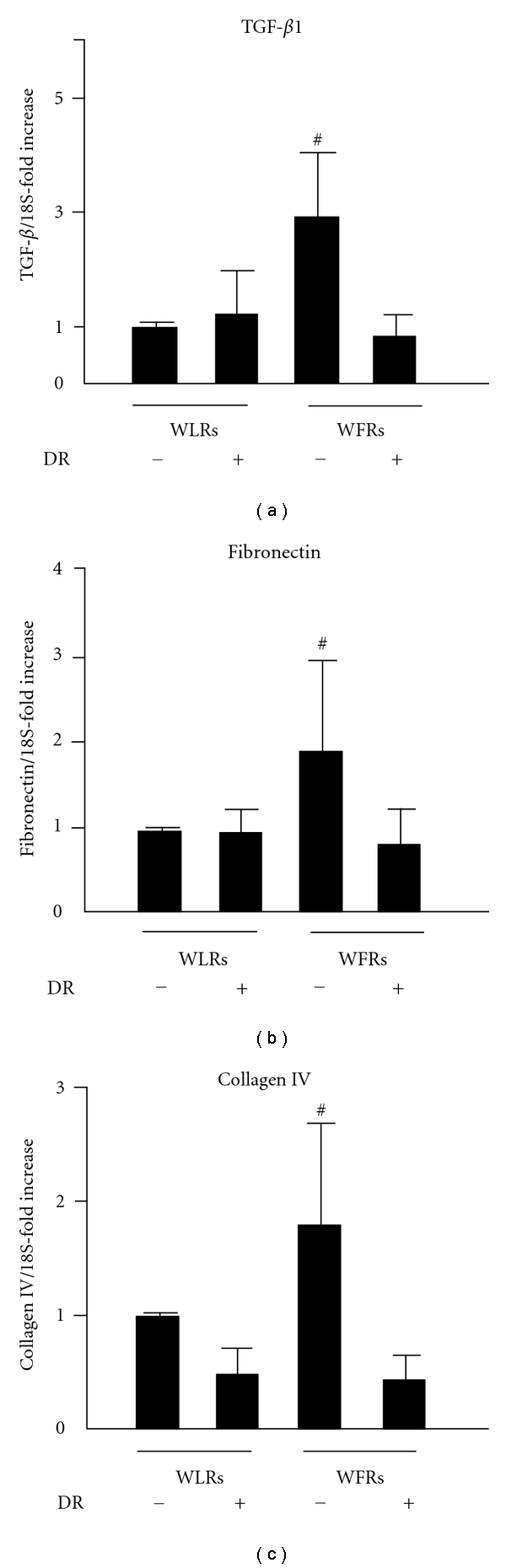
TGF-*β*, fibronectin, and collagen IV mRNA expression levels in the kidney. The mRNA expression levels of TGF-*β* (a), fibronectin (b), and collagen IV (c) were quantified using real-time PCR and expressed as fold increases from Wistar lean rats (WLRs). Data are means ± SD (*n* = 9 − 11, ^#^ *P* < 0.05 versus other groups).

We also assessed TGF-*β*1, fibronectin, and collagen IV mRNA expression levels in the kidney. The mRNA expression of these fibrosis-related genes was significantly higher in the kidneys of WFRs than that in the kidneys of WLRs (Figures 3(a)–3(c)). These alterations in WFRs were improved by DR.

### 3.5. Changes in Macrophage Infiltration in the Kidney

**Figure 4:**
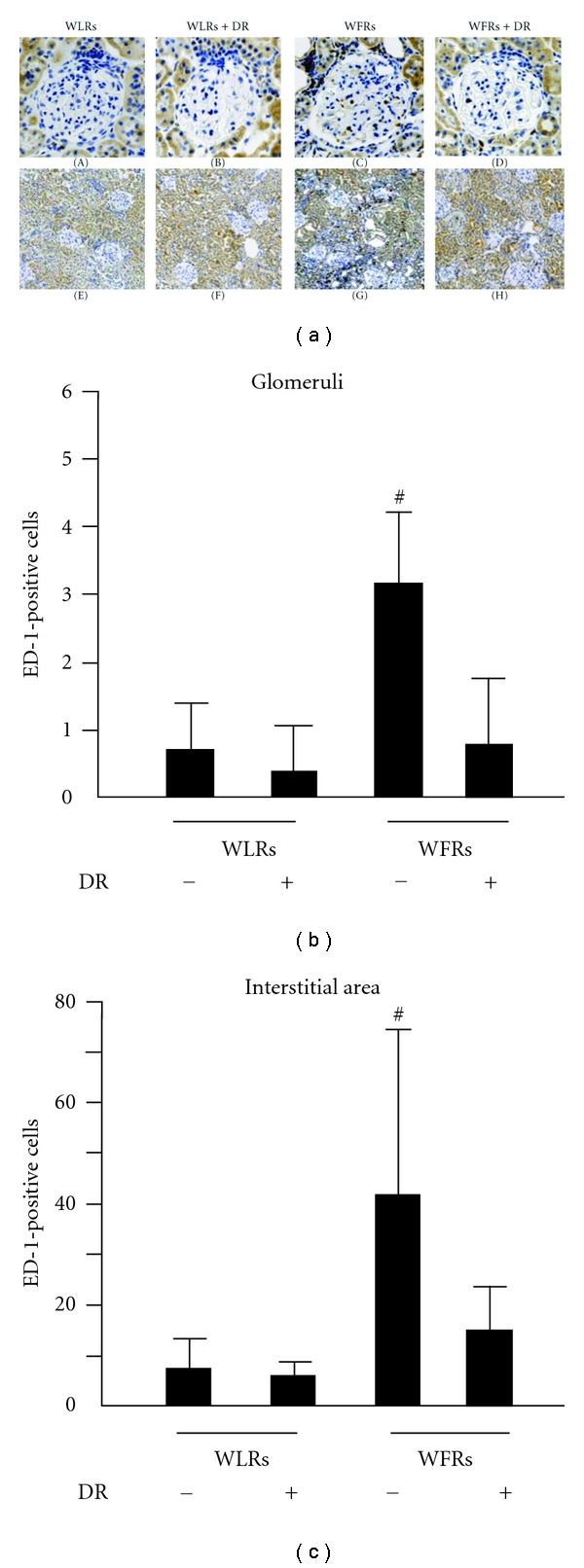
Treatment with DR suppresses the number of ED-1-positive cells in the kidney of WFRs. ((a)-(A) through (H)) Representative photomicrographs of renal ED-1-positive cells in the four groups of rats. Data are the results of independent experiments in each group with six mice per group. Original magnification, ×400 for glomerular ED-1 staining and ×100 for tubule-interstitial ED-1 staining. (b) and (c) ED-1-positive cells in glomerular and tubulointerstitial lesions. Data are means ± SD (*n* = 6, ^#^ *P* < 0.05  versus other groups, ^##^ *P* < 0.01 versus other groups).

The number of ED-1 (a macrophage marker)-positive cells in the renal glomeruli and interstitial lesions was significantly higher in WFRs than that in WLRs (Figure 4(a)—(A) through (H)). DR reduced the number of ED-1-positive cells in the renal glomeruli and interstitial lesions of WFRs.

### 3.6. mRNA Levels of MCP-1, ICAM-1, and VCAM-1 in the Kidney

**Figure 5:**
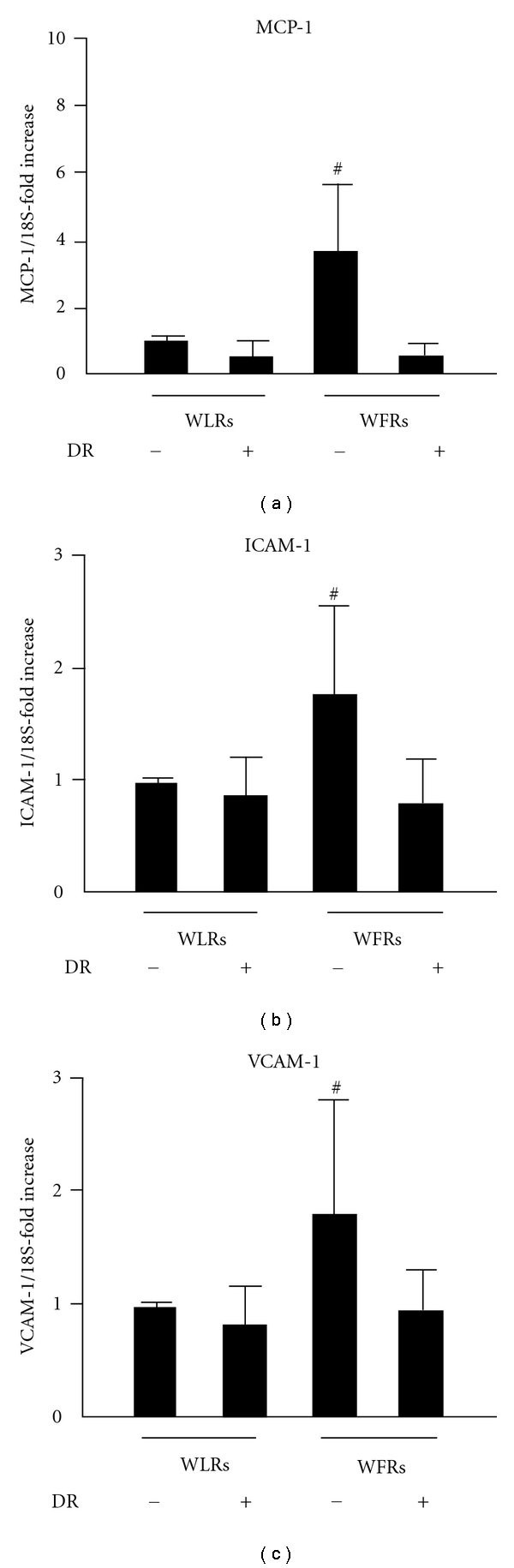
MCP-1, ICAM-1, and VCAM-1 mRNA expression levels in the kidney. The mRNA expression levels of MCP-1 (a), ICAM-1 (b), and VCAM-1 (c) were quantified using real-time PCR and expressed as fold increases from Wistar fatty rats. Data are means ± SD (*n* = 9 − 11, ^#^ *P* < 0.05 versus other groups).

We next determined the mRNA expression levels of inflammation-related genes such as MCP-1, ICAM-1, and VCAM-1 in the kidney. The mRNA expression of these genes was significantly higher in the kidneys of WFRs than those of WLRs (Figures 5(a)–5(c)). These changes in WFRs were almost completely abrogated after DR. The results in Figures 2–5 indicate that DR reduces glomerular and interstitial histological abnormalities, including mesangial expansion, renal fibrosis, and macrophage infiltration, in the kidney of WFRs.

### 3.7. Changes in the Acetylation of NF-*κ*B and Sirt 1 Protein Expression in the Kidney

**Figure 6:**
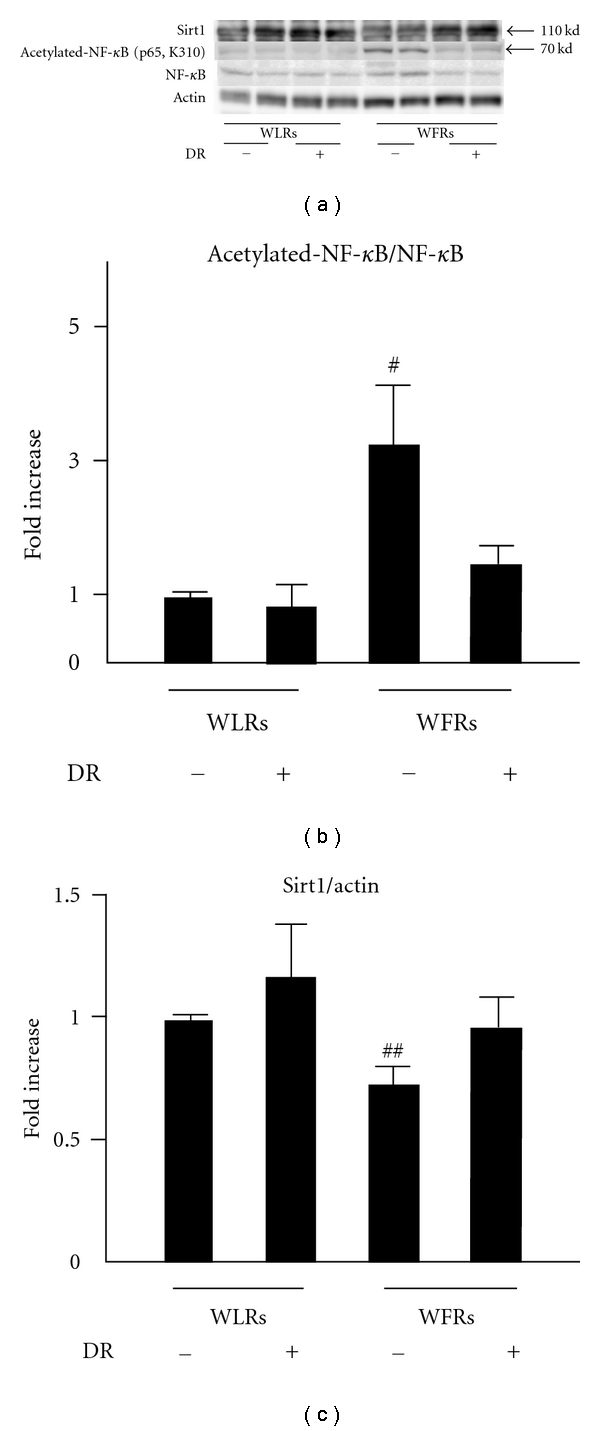
Acetylated-NF-**κ**B (p65) and Sirt1 expression in the kidney. (a) Representative immunoblots of Sirt1, acetylated-NF-**κ**B (p65), and NF-**κ**B in protein extracts from the kidneys of rats of each group. Actin was loaded as an internal control. (b) Quantitative analysis of acetylated-NF-**κ**B (p65) protein expression. (c) Quantitative analysis of Sirt1 protein expression. Data are means ± SD (*n* = 6, ^#^ *P* < 0.01 versus other groups, ^##^ *P* < 0.05 versus other groups).

We assessed the changes in acetylation of NF-*κ*B and Sirt 1 protein expression in the kidney (Figures 6(a), 6(b), and 6(c)). The level of acetylated-NF-*κ*B (p65) was significantly increased in the kidney of WFRs compared to that of WLRs. However, the protein expression of Sirt1 was decreased in the kidney of WFRs. This increase in acetylated-NF-*κ*B in the kidney of WFRs was almost completely reversed, with the level being similar to the basal levels of WLRs, after treatment with DR, which was consistent with the restoration of Sirt1 expression.

### 3.8. Changes in Autophagy in the Kidney

**Figure 7:**
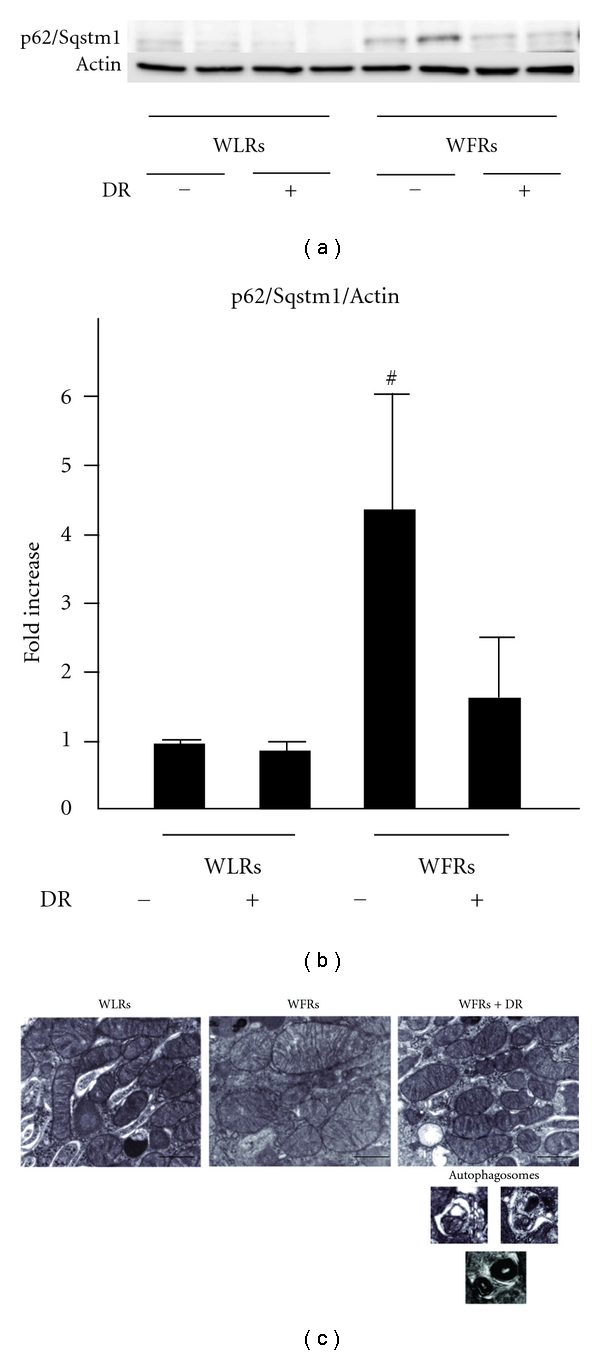
p62/Sqptm1 accumulation and electron microscopy in the kidney. (a) Representative immunoblots of p62/sequestosome 1 (Sqptm1) in protein extracts from the kidneys from rats of each group. Actin was loaded as an internal control. Data are means ± SD (*n* = 6, ^#^ *P* < 0.01 versus other groups). (b) Quantitative analysis of p62/Sqptm1expression. Data are means ± SD (*n* = 6, ^#^ *P* < 0.05 versus other groups). (c) Representative micrographs of proximal tubular cells from the, four groups of rats. Scale bar = 1 *μ*m (*n* = 3).

The accumulation of p62/Sqstm1, which is degraded through an autophagy-lysosome pathway [8], was significantly enhanced in the kidneys of WFRs (Figures 7(a) and 7(b)). Mitochondrial morphology was also altered, resulting in marked swelling and the disintegration of cristae in the renal cortex of WFRs. Under normal circumstances, damaged mitochondria are degraded by the intracellular autophagy pathway [8]. In contrast to the proximal tubular cells of WFRs, DR resulted in a restoration of abnormal mitochondrial morphology with numerous autophagosomes in WFRs (Figure 7(c)).

## 4. Discussion

In this study, we demonstrated the potential benefits of DR in ameliorating renal injuries of type 2 diabetes. DR exerted anti-inflammatory effects and improved the dysregulation of autophagy through the restoration of Sirt1 protein expression in the kidney of WFRs.

Numerous reports have shown that DR extends the lifespan of yeast, worms, flies, and mammals [3]. Recently, Colman et al. also reported that DR delayed the onset of age-associated pathologies, including diabetes and cardiovascular disease, in rhesus monkeys [4]. Moreover, Fontana et al. showed that DR in humans improved metabolism and decreased serum C-reactive protein, TNF-*α*, and carotid IMT thickening [20]. These findings suggest that the various protective effects of DR against vascular impairment are exerted through the reduction of vascular inflammation, which is strongly involved in the molecular alterations that occur in aging or age-related diseases [21]. The inflammatory process is one of the pivotal mechanisms for the initiation and progression of diabetic nephropathy and atherosclerosis [2]. However, there is little information about whether DR improves diabetic nephropathy in type 2 diabetes, especially diabetes-induced inflammation in the kidney.

First, we investigated the effects of DR on renal functional and histological abnormalities in a model of type 2 diabetes. Diabetic WFRs clearly showed increased albuminuria, a reduction in Ccr, and increased mesangial expansion and renal fibrosis. Moreover, an increase in macrophage infiltration accompanied by the overexpression of inflammation-related genes, including MCP-1, ICAM-1, and VCAM-1, was observed in the kidney of WFRs. Treatment with DR resulted in complete improvement of diabetes-induced renal injuries, including inflammation, and a partial reduction of glycated-Hb levels by approximately 20% compared to WFRs. These results indicate that the effects of DR on diabetic nephropathy may be exerted through other factors rather than its effects of an improvement of systemic metabolism such as hyperglycemia and dyslipidemia. What is the other factor? We here focused on Sirt1 as the other factor induced by DR because DR is known to significantly increase the levels of Sirt1 protein expression in most tissues, including the kidney, and the beneficial effects of DR are linked to Sirt1 activation [5, 6, 22]. 

Therefore, we asked whether Sirt1 protein expression was altered in the kidney of WFRs. Sirt1 protein expression was significantly decreased in the kidney of WFRs as compared to WLRs, and this alteration of Sirt1 expression was restored by treatment with DR. Sirt1 has anti-inflammatory properties through the deacetylation of NF-*κ*B (p65) [23, 24], which plays a central role in the regulation of the expression of inflammation-related genes, such as MCP-1, ICAM-1, and VCAM-1. Several reports have also shown that reduced levels of Sirt1 lead to the upregulation of acetylated-NF-*κ*B (p65), resulting in an increase in inflammation in the adipose tissue of high-fat diet-induced obese mice [25] or the monocytes of patients with chronic obstructive pulmonary disease (COPD) related to smoking [26]. In the present study, we showed that acetylated-NF-*κ*B (p65) was clearly increased in the kidney of WFRs compared to WLRs, and this alteration of acetylated-NF-*κ*B (p65) in WFRs was lessened by DR. This result is consistent with the restoration of Sirt1 protein expression. The p65 subunit of NF-*κ*B selectively interacts with Sirt1, which promotes the deacetylation of the p65 subunit and leads to the attenuation of the transcriptional activity of NF-*κ*B and inflammation [23]. Therefore, our results indicate that renal inflammation was induced by increased levels of acetylated-NF-*κ*B (p65) owing to the reduced levels of Sirt1 protein expression, and DR exerted anti-inflammatory effects through the restoration of Sirt1 expression in the kidney of WFRs. 

Autophagy is a lysosomal degradation pathway in cellss and plays a crucial role in removing protein aggregates and damaged or excess organelles, such as mitochondria, to maintain intracellular homeostasis and maintain the cell health under various stress conditions [8, 9]. The renoprotective role of autophagy has been shown in several animal experimental models, such as aging [10] and acute kidney injuries [11–14]. In the present study, we found that mitochondrial morphological damages in the proximal tubular cells and p62/Sqstm1 accumulation in diabetic kidneys of WFRs occurred, which suggested that an impairment of autophagy system induced mitochondrial damage. Because Sirt1 is one of the positive regulators of autophagy, the decrease in Sirt1 expression observed in the diabetic kidney may lead to the dysregulation of autophagy. In addition, the phosphorylation of AMPK, which positively regulates Sirt1 activity [27, 28] and autophagy [8], was also decreased in the diabetic kidney (data not shown). DR improved the function of autophagy system, which resulted in normalization of mitochondrial morphological changes and p62/Sqstm1 accumulation accompanied by the restoration of Sirt1 expression and AMPK activation in the kidney of WFRs. We also confirmed the existence of numerous autophagosomes in the proximal tubular cells of the kidney in WFRs treated with DR, consistent with the existence of normal morphological mitochondria. In addition, when autophagy is inhibited, p62/Sqstm1 accumulation alters the NF-*κ*B pathway and leads to inflammation [29, 30]. These data from previous reports suggest that p62/Sqstm1 accumulation associated with the dysregulation of autophagy is involved in increased inflammation. However, the role of autophagy in the pathogenesis of diabetic nephropathy and the relationship between inflammation and dysregulation of autophagy are still unclear. Therefore, further study will be needed to clarify these mechanisms in detail.

## 5. Conclusions

The current study demonstrated the beneficial effects of DR on renal injuries, including inflammation, in type 2 diabetic nephropathy model. The decrease in Sirt1 protein expression in the diabetic kidney may lead to inflammation through increased levels of acetylated-NF-*κ*B (p65) and through the dysregulation of autophagy. DR may ameliorate the inflammation and the dysregulation of autophagy via the restoration of Sirt1 protein expression in the diabetic kidney. Therefore, Sirt1 may be a significant therapeutic target for the prevention of nephropathy in type 2 diabetes.

##  Conflict of Interests

The authors declare no conflict of interests.

## Figures and Tables

**Table 1:** Effects of DR on body weight, kidney weight, blood pressure, blood glucose, glycated-Hb, and lipid profiles in the four groups of rats.

	WLRs (*n* = 10)	WLRs + DR (*n* = 11)	WFRs (*n* = 10)	WFRs + DR (*n* = 9)
Body weight (g)	426.3 ± 31.4	264.2 ± 32.7^a^	671.3 ± 70.3^a^	489.8 ± 34.8^b^
Kidney weight (g)	2.8 ± 0.17	1.8 ± 0.06^a^	3.0 ± 0.35^a^	2.23 ± 0.91^a^
Systolic blood pressure (mmHg)	121.9 ± 6.8	113.0 ± 11.0	126.3 ± 15.4	120.3 ± 5.1
Fasting blood glucose (mg/dL)	96.7 ± 8.8	95.7 ± 5.6	111.1 ± 11.9^c^	115.4 ± 6.1^c^
Glycated-Hb (%)	3.1 ± 0.25	3.1 ± 0.17	6.1 ± 0.56^c^	4.9 ± 0.9^c, d^
Total cholesterol (mg/dL)	132.2 ± 14.6	88.4 ± 6.7^a^	207 ± 32.3^c^	164.0 ± 26.5^b^
Triglyceride (mg/dL)	25.3 ± 4.9	20.5 ± 10.0	179.4 ± 93.3^c^	77.4 ± 25.5^c, d^

Data are means ± SD.

Data are means ± SD ^a^ *P* < 0.01 versus other groups, ^b^ *P* < 0.05 versus other groups, ^c^ *P* < 0.01 versus WLRs, WLRs + CR, ^d^ *P* < 0.05 versus WFRs.
